# ﻿Description of the larva of Cybister (Melanectes) brevis Aubé, 1838 (Coleoptera, Dytiscidae, Cybistrinae)

**DOI:** 10.3897/zookeys.1257.168982

**Published:** 2025-10-29

**Authors:** Kohei Watanabe, Masakazu Hayashi

**Affiliations:** 1 Ishikawa Insect Museum, Hakusan, Ishikawa, 920–2113 Japan Ishikawa Insect Museum Ishikawa Japan; 2 Hoshizaki Green Foundation, Izumo, Shimane, 691–0076 Japan Hoshizaki Green Foundation Izumo Japan

**Keywords:** Chaetotaxy, diving beetle, larval stage, Red List, water beetle

## Abstract

Cybister (Melanectes) brevis Aubé, 1838 is an endangered species in Japan. Here, we present the first description of C. (M.) brevis larvae, emphasizing the chaetotaxy of the cephalic capsule, head appendages, legs, last abdominal segment, and urogomphi. Morphological differences between the subgenera *Cybister* Curtis, 1827 and *Melanectes* Brinck, 1945 include (1) the width of notches between medial and lateral projections of frontoclypeus, (2) the shape of egg bursters, and (3) the appearance of the clypeal lamellae.

## ﻿Introduction

Study of larval morphology of the beetle family Dytiscidae Leach, 1815 has garnered increasing attention since the 1990s whilst emphasis was put on chaetotaxy and porotaxy analyses (Alarie and Michat 2023). Such descriptive approach resulted in remarkable advancements allowing discovery of new character sets that have enhanced comparisons among taxa.

Cybistrinae Sharp, 1880 are large to very large-bodied Dytiscidae, comprising 12 genera worldwide (Miller et al. 2024; Nilsson and Hájek 2025). Larval descriptions have been recorded for 9 of these 12 genera—*Bifurcitus* Brinck, 1945, *Cybister* Curtis, 1827, *Megadytes* Sharp, 1882, *Metaxydytes* Miller, Michat & Ferreira, 2024, *Onychohydrus* Schaum & White, 1847, *Paramegadytes* Trémouilles & Bachmann, 1980, *Spencerhydrus* Sharp, 1882, *Sternhydrus*, Brinck, 1945, and *Trifurcitus* Brinck, 1945 (Miller et al. 2024). This paper is meant to be a contribution towards the study of the larval morphology of the genus *Cybister*, which is comprised of four subgenera: *Cybister* Curtis, 1827, *Megadytoides* Brinck, 1945, *Melanectes* Brinck, 1945, and *Neocybister* Miller, Bergsten & Whiting, 2007 (Nilsson and Hájek 2025). At the present time, larval descriptions are only available for selected members of the subgenus Cybister (Fiori 1949; Watts 1964; Alarie et al. 2011; Watanabe and Hayashi 2024). More specifically, this contribution aims at formally describing the larvae of a species of the subgenus Melanectes, namely that of Cybister (M.) brevis Aubé, 1838, which is distributed across Japan, China, Taiwan, and South Korea (Nakajima et al. 2020; Watanabe and Yoshitomi 2022; Jiang et al. 2023). Whereas previously superficially described (Ichikawa 1984; Kamite 2008; Mitamura et al. 2017; Inoda et al. 2022; Watanabe and Hayashi 2023; Watanabe, 2024), this species would benefit from being described according to the new descriptive format including chaetotaxy and porotaxy analyses (Alarie and Michat 2023) thereof facilitating comparison with larvae of other known Cybistrinae larvae. It is worth noting that Cybister (M.) brevis is listed as “Near Threatened” on the Japanese Red List (Ministry of the Environment of Japan 2015).

## ﻿Materials and methods

Seven larvae used for the description were obtained through rearing, following the methods described by Watanabe et al. (2021, 2023). An additional 10 specimens were collected in the field. The larvae were fixed in 70% ethanol and subsequently mounted on slides in 70% ethanol or on HS-slides (Shirayama et al. 1993) with Euparal. The specimens were observed under an optical microscope (Nikon ECLIPSE E400) up to 400× magnification and sketched using a Nikon Y-IDT drawing tube. After scanning the sketches, line drawings were prepared using an iPad Pro 11-inch (4^th^ generation). Photographs of the living larvae were captured using a Nikon D500 digital camera equipped with a Nikon AF-S Micro NIKKOR 60 mm f/2.8G ED lens. Measurements were obtained using a stereomicroscope (Leica M205C, Planapo 1.0×) with a transmitted light base (Leica TL3000 Ergo), a camera (Leica DFC420), and LAS software (v. 3.3.1). Fine structural details of the specimens were observed using a scanning electron microscope (SEM; JEOL JCM-6000 Neoscope Scanning Electron). The larvae were freeze-dried and coated with ultrathin gold layers using high-vacuum evaporation. The examined larvae have been deposited in the larval collections of the Ishikawa Insect Museum (**IIM**), Ishikawa, Japan, the Kohei Watanabe Collection (**KWC**), Ishikawa, Japan, and the Hoshizaki Green Foundation (**HOWP**), Shimane, Japan. The observation methods used in the study followed Watanabe and Hayashi (2024). The measurements and notation of primary setae and pores used in this study follow Alarie et al. (2011), Michat et al. (2015, 2019), and Watanabe and Hayashi (2024). The terms: **A** and **AN**, antenna; **AB** and **LAS**, abdominal segment VIII (last abdominal segment); **CL**, longest claw; **CO**, coxa; **COL**, coronal line length; **FE**, femur; **FR**, frontoclypeus; **FRL**, frontoclypeus length; **HL**, head length; **HW**, maximum head width; **L**, leg; **LA**, labium; **LP**, labial palpi; **MN**, mandible; **MNL**, mandible length; **MNW**, mandible width; **MP**, maxillary palpi; **MX**, maxilla; **OCW**, occipital foramen width; **PA**, parietal; **PPF**, maxillary palpifer; **PT**, pretarsus; **TA**, tarsus; **TI**, tibia; **TL**, total length; **TR**, trochanter; **U** and **UR**, urogomphus.

## ﻿Results

### ﻿Description of the larvae of Cybister (Melanectes) brevis Aubé, 1838

#### ﻿ Cybister (Melanectes) brevis

Taxon classificationAnimaliaColeopteraDytiscidae

Aubé, 1838

E0C191E6-6732-5A1E-84C1-B9E2723A38A3

Figs 1–5, 6, 7, 8–10, 11–14, 15, 16, 17–19, 20–25, 26–31, 32–33, Table 1

##### Source of material.

Descriptions were based on five instar I and two instar III specimens (HOWP, IIM) reared *ex ovo* in the laboratory at the Ishikawa Insect Museum from adults collected at the following locality: Japan • Hakusan-shi, Ishikawa Prefecture; 21.VI.2023; K. Watanabe leg. Additional specimens were collected in association with adults at the following localities: Japan • three instar I (KWC); Awazu, Misaki-machi, Suzu-shi, Ishikawa Prefecture; 9.VII.2023; K. Watanabe leg.; • one instar II (KWC); Araya-machi, Nomi-shi, Ishikawa Prefecture; 24.VI.2021; K. Watanabe leg.; • one instar I, two instar II (KWC); Yawata-machi, Hakusan-shi, Ishikawa Prefecture; 21.VI.2023; K. Watanabe leg.; • two instar II (KWC); Fushimi, Misaki-machi, Suzu-shi, Ishikawa Prefecture; 25.VI.2023; K. Watanabe leg.; • one instar III (KWC); idem; 9.VII.2023; K. Watanabe leg.

**Figures 1–5.**
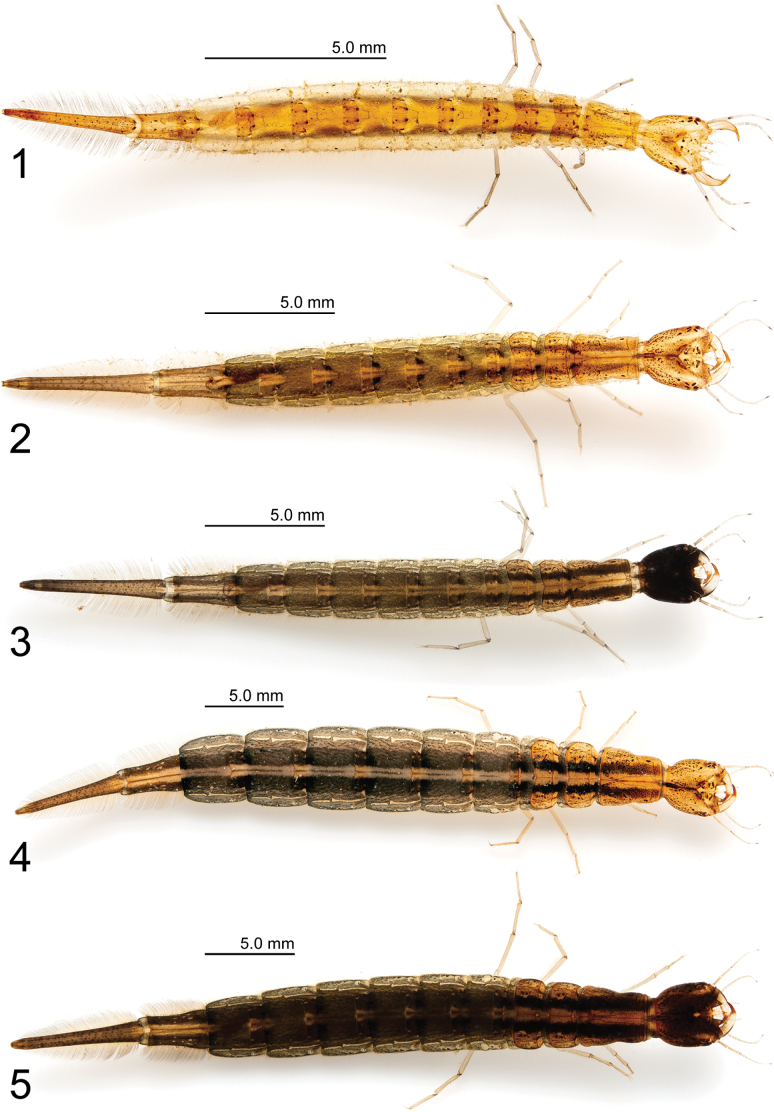
Cybister (Melanectes) brevis Aubé, 1838. 1. Instar I; 2, 3. Instar II; 4, 5. Instar III.

##### Diagnosis.

Larvae of C. (M.) brevis can be distinguished from those of other *Cybister* species by the following combination of characteristics: egg burster spiniform; lamellae clypeales with weak longitudinal unevenness and split into several hair-like projections; lateral projections of frontoclypeus narrow; proximal article of A2 dark brown; distal article of A1 approximately < 1.3 times longer than basal article; primary seta AN3 absent; some individuals with almost entirely black (instar II) or dark brown (instar III) head capsule; pronotum with two dark-brown longitudinal stripes dorsally (instars II and III).

##### Description.

**Figures 6, 7.**
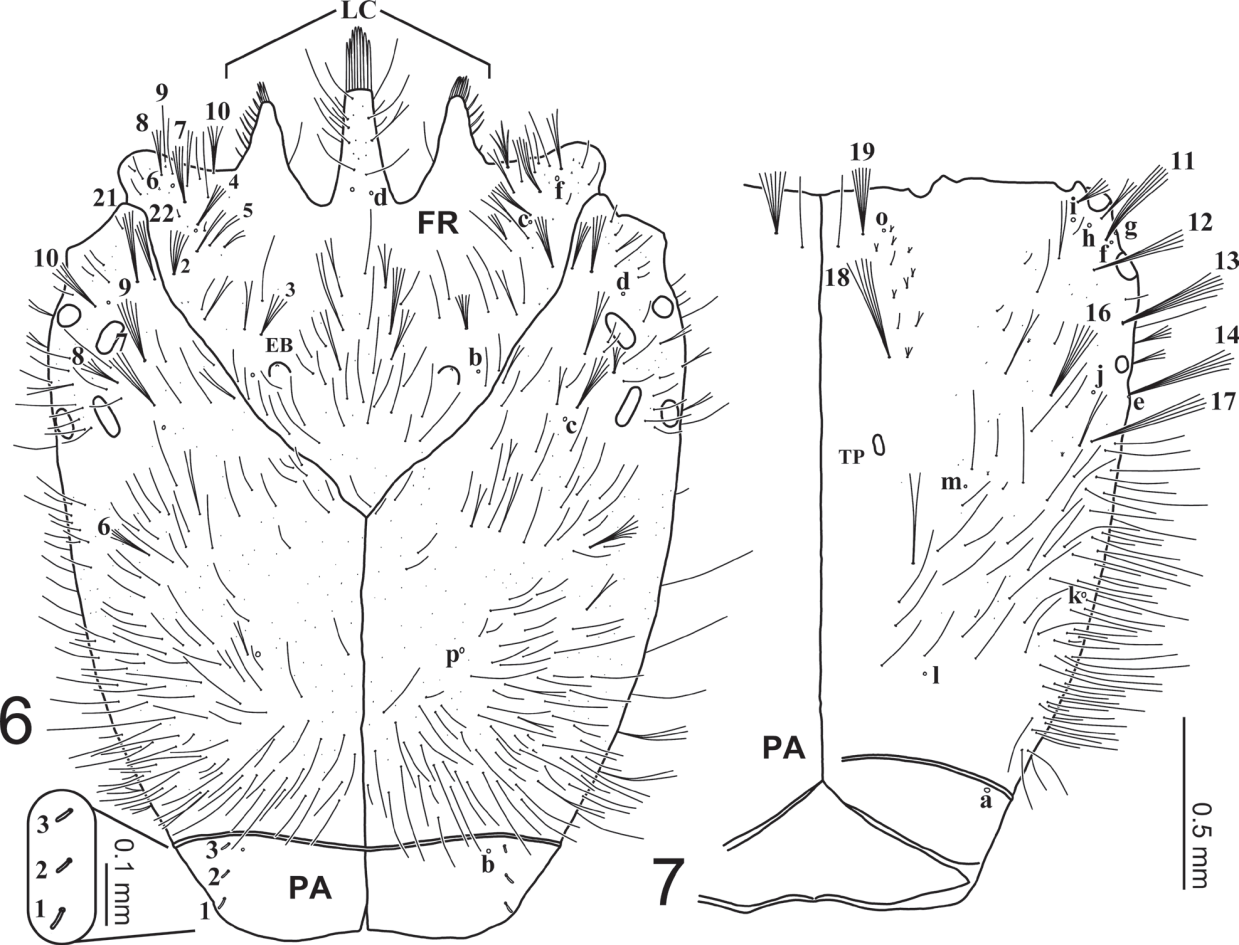
First-instar larva of Cybister (Melanectes) brevis Aubé, 1838, cephalic capsule. 6. Dorsal aspect; 7. Ventral aspect. EB, egg burster; LC, lamellae clypeales; TP, tentorial pit.

**Figures 8–10.**
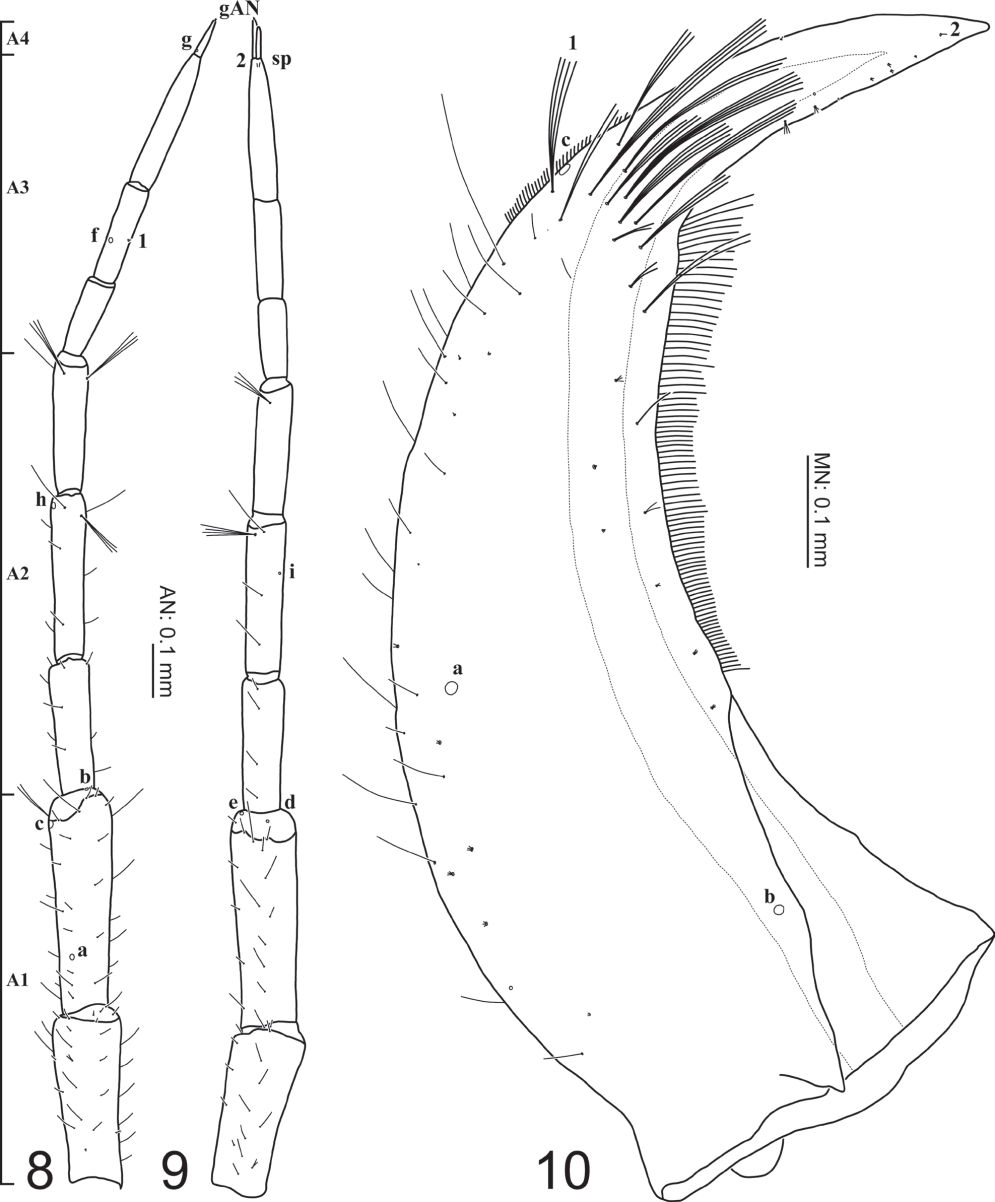
First-instar larva of Cybister (Melanectes) brevis Aubé, 1838. 8, 9. Antenna; 10. Mandible; 8, 10. Dorsal aspect; 9. Ventral aspect. sp, spinula.

**Figures 11–14.**
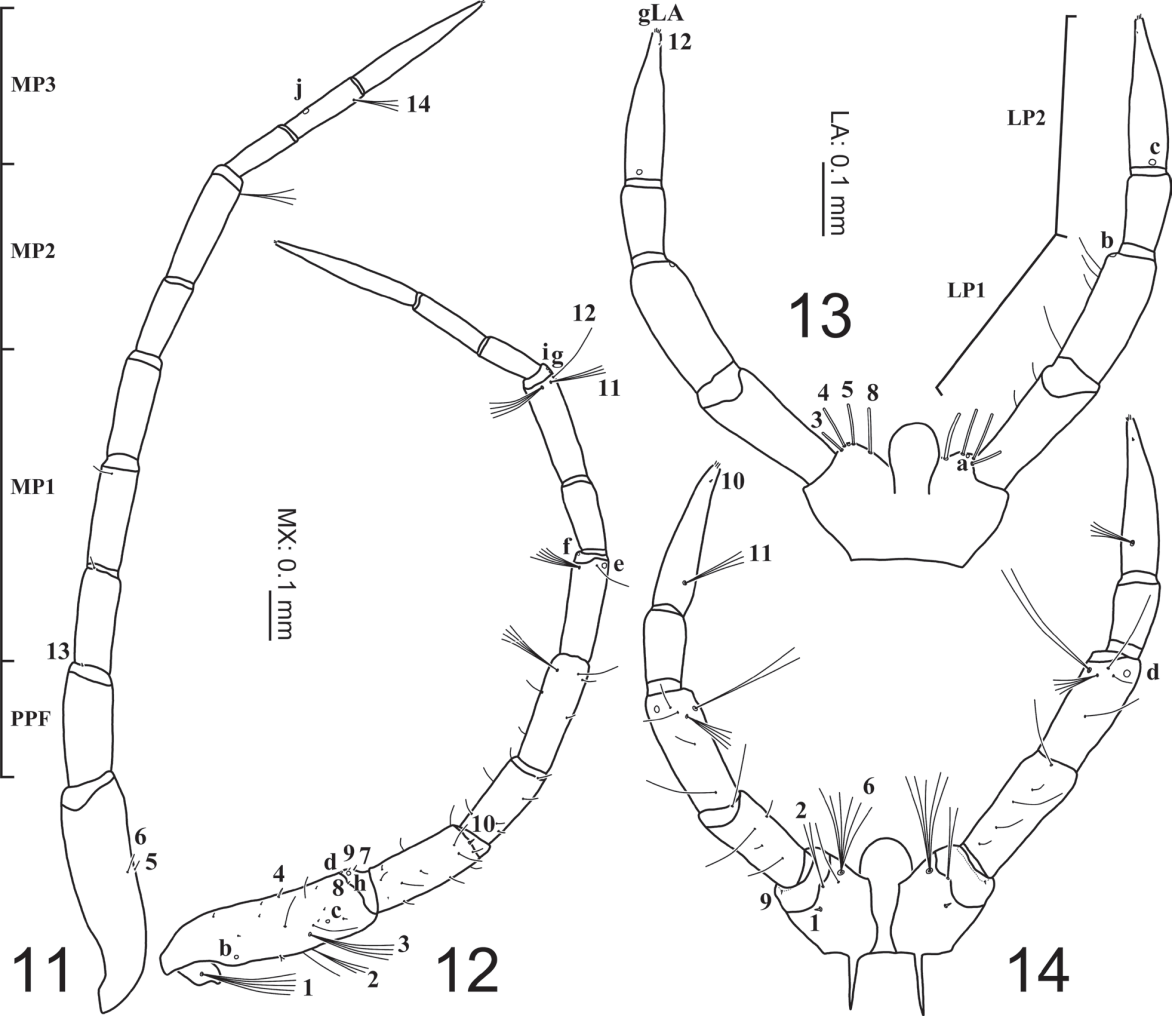
First-instar larva of Cybister (Melanectes) brevis Aubé, 1838. 11, 12. Maxilla; 13, 14. Labium; 11, 13. Dorsal aspect; 12, 14. Ventral aspect.

**Figures 15, 16.**
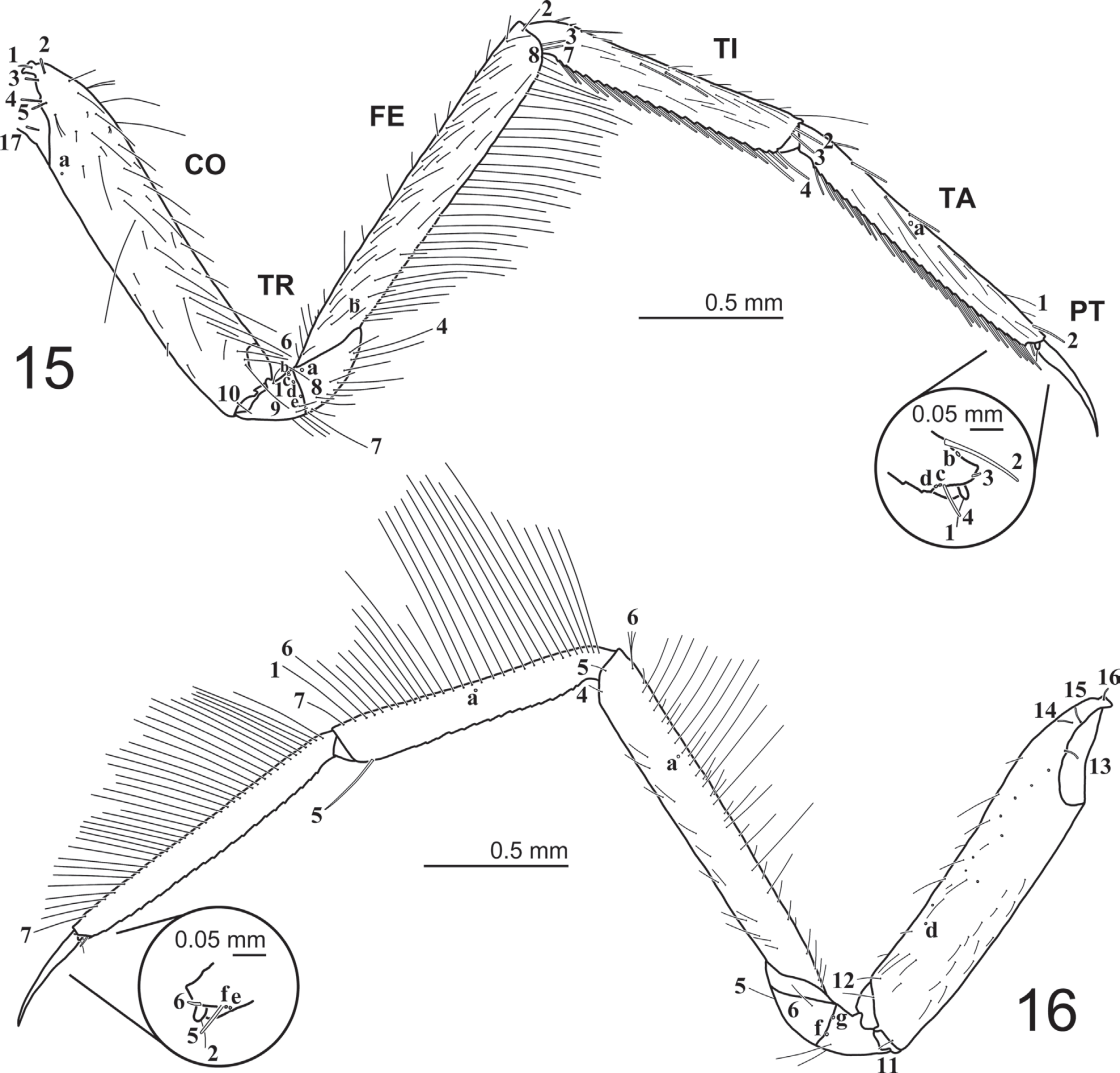
First-instar larva of Cybister (Melanectes) brevis Aubé, 1838, metathoracic leg. 15. Anterior aspect; 16. Posterior aspect.

**Figures 17–19.**
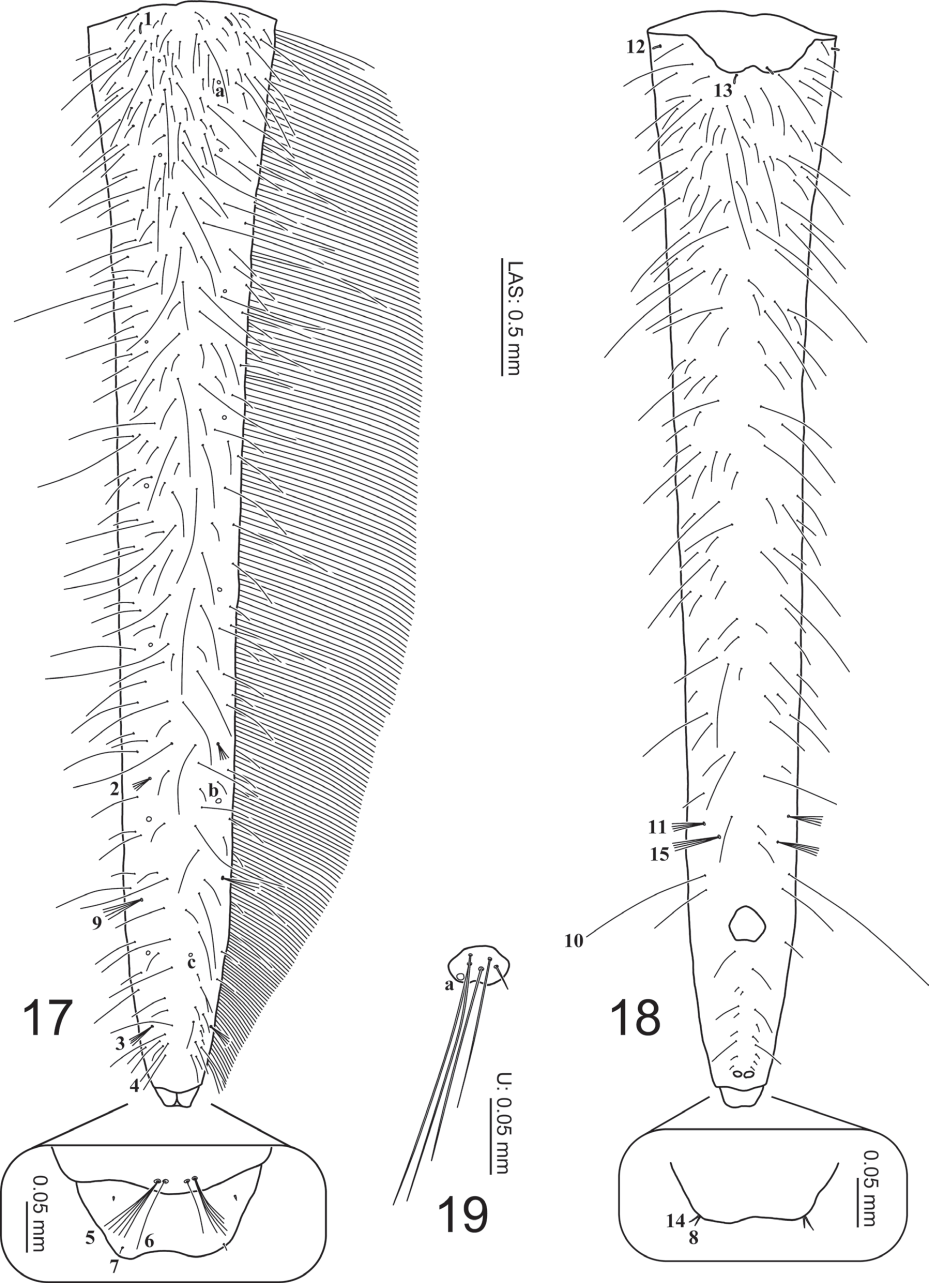
First-instar larva of Cybister (Melanectes) brevis Aubé, 1838. 17, 18. Abdominal segment VIII; 19. Urogomphus; 17. Dorsal aspect; 18, 19. Ventral aspect.

**Figures 20–25.**
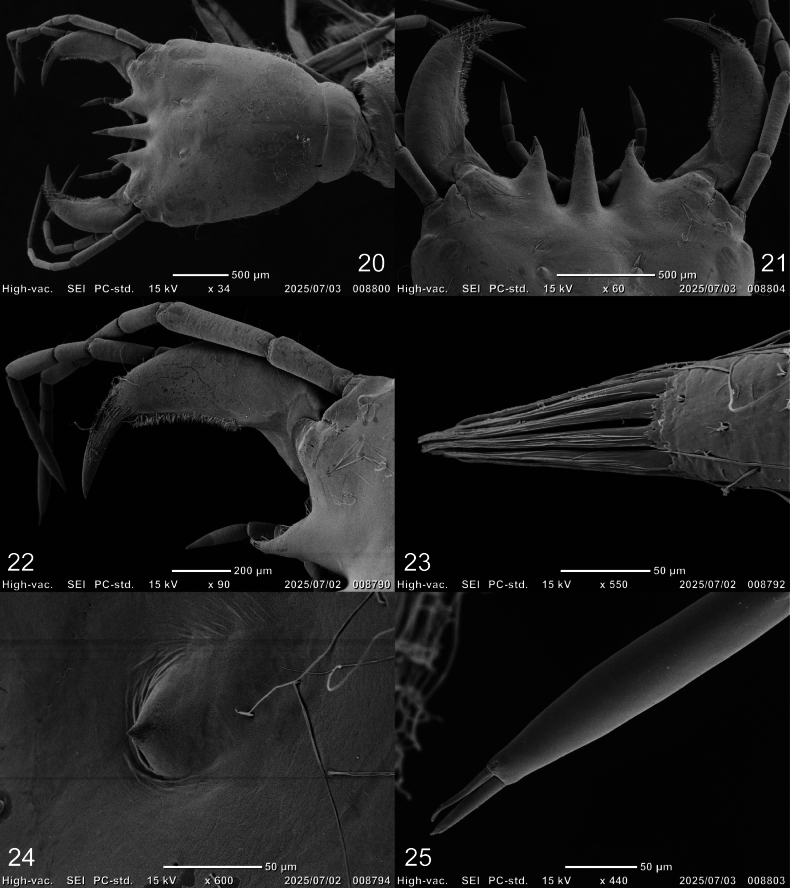
SEM photographs of first-instar larva of Cybister (Melanectes) brevis Aubé, 1838, head. 20. Cephalic capsule; 21, 22. Mandible and projections of lamellae clypeales; 23. Lamellae clypeales of central projection; 24. Egg burster; 25. A3, A3’, and A4, ventral aspect.

**Figures 26–31.**
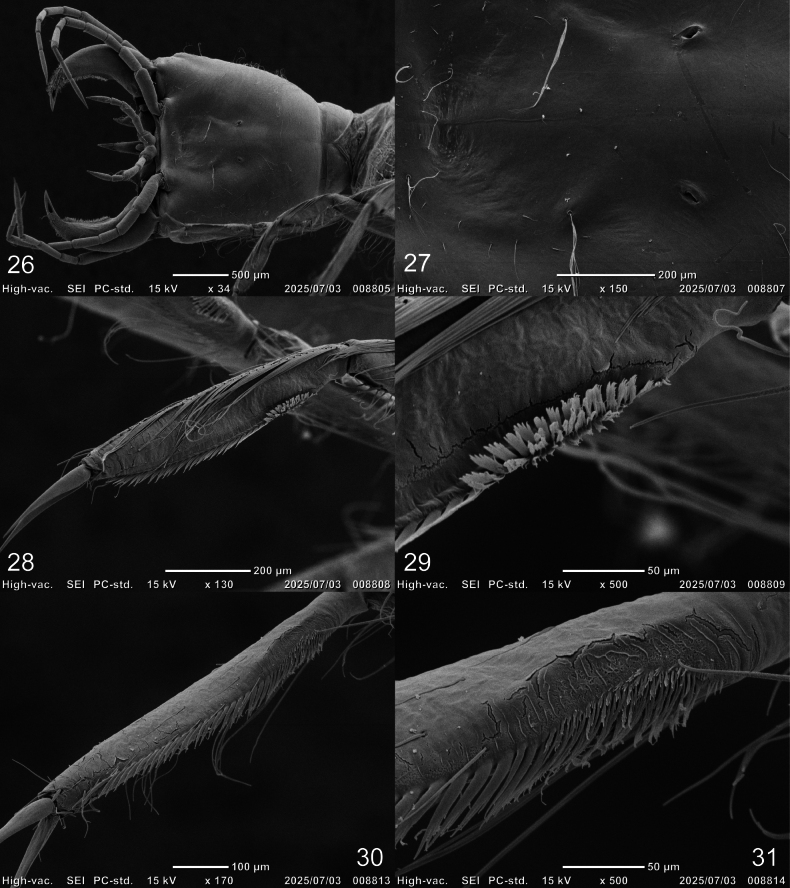
SEM photographs of first-instar larva of Cybister (Melanectes) brevis Aubé, 1838. 26. Cephalic capsule; 27. Tentorial pit; 28–31. Tarsus of prothoracic leg; 26, 27. Ventral aspect; 28, 29. Posterior aspect; 30, 31. Anterior aspect.

**Instar I (Figs 1, 6–33). *Color* (Fig. 1).** Head capsule yellow-brown; frontoclypeus and parietal region internal to stemmata lighter; with several small brown maculae; stemmata brown; antennae pale yellow except for first article of A2, apical half of third article of A3 and A4 dark brown; mandible pale yellow except for distal portion light yellow to red-brown, thick row of small setae on inner margin brown; maxilla pale yellow except for third article of palpomere 3 dark brown; labium pale yellow; thoracic tergites yellow-brown with several small brown maculae and pair of slightly larger brown maculae on anterior quarter of protergite; prosternal sclerites yellow-brown; base of setae on each posterior side of membranous region of meso- and metathorax brown; abdominal tergites I–VI yellow-brown with several small brown maculae; base of setae on lateromedial and ventral membranous regions brown; abdominal tergites VII–VIII yellow-brown with several small brown maculae; base of setae on ventral membranous region of abdominal tergite VII brown; legs pale yellow; urogomphus yellow-brown.

***Body* (Fig. 1).** Elongate, subcylindrical; measurements and body shape ratios are shown in Table 1.

**Table 1.** Measurements and ratios for the larvae of Cybister (Melanectes) brevis Aubé, 1838. *N* = number of specimens examined.

Measure	C. brevis			Measure	C. brevis		
	Instar I (N = 3)	Instar II (N = 3)	Instar III (N = 3)		Instar I (N = 3)	Instar II (N = 3)	Instar III (N = 3)
HL (mm)	2.33–2.38	3.46–3.54	4.84–5.15	PPF/MP1	0.33–0.36	0.41–0.46	0.46–0.48
HW (mm)	1.63–1.65	2.49–2.52	3.50–3.70	MP1/MP2	1.68–1.75	1.62–1.80	1.69–1.88
FRL (mm)	1.07–1.09	1.48–1.53	1.88–1.98	MP3/MP2	1.52–1.59	1.31–1.37	1.24–1.29
OCW (mm)	0.70–0.76	1.19–1.26	1.84–2.04	MP/LP	1.95–2.10	2.04–2.16	1.68–1.94
HL/HW	1.43–1.45	1.39–1.41	1.36–1.39	LP2/LP1	0.69–0.81	0.71	0.51–0.60
HW/OCW	2.17–2.34	2.00–2.10	1.80–1.90	L3 (mm)	4.84–4.94	7.17–7.35	9.46–9.79
COL/HL	0.53–0.55	0.57–0.58	0.61–0.62	L3/L1	1.26–1.30	1.28–1.29	1.26–1.30
FRL/HL	0.45–0.47	0.42–0.43	0.38–0.39	L3/L2	1.13–1.14	1.14–1.15	1.12–1.16
A/HW	1.18–1.22	1.06–1.08	0.91–0.98	L3/HW	2.94–3.03	2.84–2.93	2.59–2.80
A2/A1	1.01–1.05	0.94–0.95	0.78–0.88	L3 (CO/FE)	1.00–1.14	0.99–1.02	0.97–0.98
A3/A1	0.71–0.74	0.60–0.67	0.50–0.54	L3 (TI/FE)	0.69–0.78	0.70–0.72	0.66–0.68
A4/A3	0.13–0.14	0.10–0.11	0.08–0.09	L3 (TA/FE)	0.81–0.89	0.71–0.73	0.63–0.64
A3’/A4	0.63–0.79	0.79–0.88	0.72–0.85	L3 (CL/TA)	0.35–0.36	0.32–0.33	0.26–0.29
MNL/MNW	2.75–2.87	2.73–2.81	2.84–2.92	LAS (mm)	4.05–4.13	5.85–5.94	7.44–7.58
MNL/HL	0.43–0.46	0.44	0.42–0.45	LAS/HW	2.46–2.51	2.34–2.37	2.04–2.12
A/MP	1.31–1.39	1.36–1.38	1.31–1.36	U (mm)	0.02	0.03	0.06–0.07

***Head* (Figs 6–14, 20–27).***Cephalic capsule* (Figs 6, 7, 20, 26). Flattened, subquadrate, longer than broad; maximum width at level of anterior stemmata, constricted at level of occipital region (Fig. 20), lacking temporal spiniform setae; occipital suture present; ecdysial line well marked, COL long; occipital foramen deeply emarginate ventrally (Figs 7, 26); tentorial pits visible slightly above middle of ventral midline (Figs 7, 26, 27); frontoclypeus subtriangular, anterior margin projected forward, divided into three well-developed projections, medial projection slightly longer than lateral projections, medial projection triangular, narrow and of same length on both sides, lateral projections triangular, slightly broader than medial projection, with inner length longer than outer length, inner margin slightly curved, notches between medial and lateral projections wide; anterolateral lobes rounded, not projecting beyond lamellae clypeales; EB present, large, spiniform (Figs 6, 24), near ecdysial line at level of seta PA8; six stemmata on each side, four dorsal, two ventral. *Antenna* (Figs 8, 9, 25). Elongate, slender, slightly longer than HW, composed of four antennomeres; A1 and A2 subequal in length, A1 subdivided into two articles, distal article approximately 1.3 times longer than basal article; A2 subdivided into three articles: first article shortest, slightly shorter than third article, second article longest; A3 shorter than A2, subdivided into three articles: first article shortest, slightly shorter than second article, third article longest; A3’ elongate, slender, slightly shorter than A4; A4 shortest. *Mandible* (Figs 10, 21, 22). Strong, falciform, broadest at base, narrowing to apex, abruptly narrowed toward apex from pore MNc; mandibular channel present. *Maxilla* (Figs 11, 12, 26). Premaxillary lobes well developed; cardo well developed, subovate with slightly concave apex, stipes elongate, slender, subcylindrical; galea absent; PPF elongate, slender, palpomere-like; MP elongate, slender, shorter than antenna, composed of three palpomeres, MP1 longest, MP2 shortest; MP1 subdivided into three subequal articles, MP2 subdivided into two articles, distal article longer than basal article; MP3 subdivided into three articles, first and second articles subequal in length, third article longest. *Labium* (Figs 13, 14, 26). Prementum broader than long, not sclerotized ventromedially, anterodorsal margin well projected forward into rounded median process; LP approximately half as long as MP, composed of two palpomeres; LP1 longer than LP2; LP1 subdivided into two articles subequal in length; LP2 subdivided into two articles, distal article longer than basal article.

***Thorax* (Figs 1, 15, 16, 28–31).** Pro-, meso-, and metanotum convex, with subequal width; protergite longer than broad, twice as long as mesotergite, subrectangular, lateral margins emarginate at about mid-length, anterior and posterior margins straight; meso- and metatergite small, broader than long, subequal in length, subtrapezoidal, posterior margin emarginate medially; sagittal line present on all tergites; sternum of prothorax membranous except for one pair of small subtriangular sclerites, sterna of meso- and metathorax membranous; spiracles absent. Legs (Figs 15, 16, 28–31). Long, composed of six segments, L1 shortest, L3 longest; CO, FE, TI, and TA subcylindrical, elongate, slender; TR short, divided into two parts by an annulus; PT with two long, curved claws; TA of L1 with apically multifid setae forming a dense patch (cleaning device), these setae larger and flat on posteroventral region (Figs 28, 29), smaller on anteroventral region (Figs 30, 31).

**Figures 32, 33.**
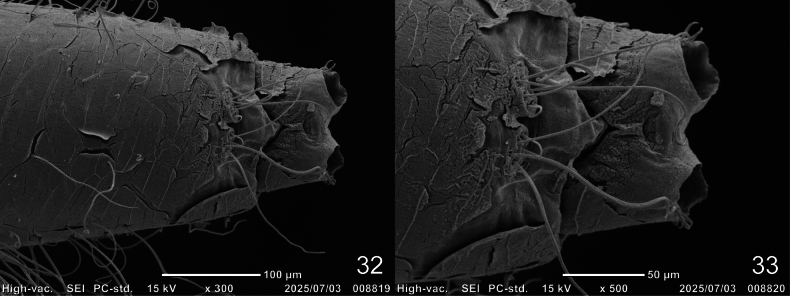
SEM photographs of first-instar larva of Cybister (Melanectes) brevis Aubé, 1838, abdominal segment VIII and urogomphus, ventral aspect.

***Abdomen* (Figs 1, 17–19, 32, 33).** Eight-segmented; segments I–VI subequal in length, mostly membranous with a small tergite on anterodorsal region, tergites I–VI subrectangular, without anterior carina, sagittal line present; sterna of segments I–VI membranous; segment VII narrower, subtrapezoidal, without anterior carina, sagittal line absent, fully sclerotized except ventrally; segments I–VII without spiracles; segment VIII longest and narrowest, fully sclerotized except around anus. Urogomphus (Figs 19, 32, 33). Strongly reduced in length, slightly broader than long, comprised of one urogomphomere.

***Chaetotaxy*.** Similar to that of generalized *Cybister* larva (Alarie et al. 2011; Alarie and Michat 2023; Watanabe and Hayashi 2024) with the following remarks: lamellae clypeales with weak longitudinal unevenness and split into several hair-like projections (Fig. 23); setae PA1–3 apically rounded (Fig. 6); seta FR10 broad (Fig. 6); seta AN3 absent (Figs 9, 25); row of short setae present on outer margin of mandible, around pore MNc (Fig. 10); setae MX11 and MX14 multi-branched (Figs 11, 12); seta TR4 not multi-branched (Fig. 15); setae AB1, AB12, and AB13 apically rounded (Figs 17, 18); seta AB4 long (Fig. 17).

**Instar II (Figs 2, 3).** As for instar I except as follows:

***Color* (Figs 2, 3).** Head capsule varies among individuals, ranging from yellow-brown with numerous small brown maculae (Fig. 2) to almost entirely blackened (except for frontoclypeal anterior projections and area posterior to occipital suture) (Fig. 3); thoracic tergites and abdominal tergites I–VI with two brown to dark brown longitudinal stripes; membranous region of pro-, meso-, metanotum, and abdominal segments I–VI gray-brown, paler on both sides; abdominal tergite VII light-brown to gray-brown on both sides, yellow-brown in center with several small brown maculae; abdominal tergite VIII light brown to gray-brown with several small brown maculae.

***Body*.** Measurements and body shape ratios are shown in Table 1.

***Head*.** EB absent; HW/OCW = 2.00–2.10. A/HW = 1.06–1.08; A2/A1 = 0.94–0.95; A3/A1 = 0.60–0.67. MP3/MP2 = 1.31–1.37.

***Chaetotax*y.** Identification of secondary setae was difficult due to the large number of additional setae.

**Instar III (Figs 4, 5).** As for instar II except as follows:

***Color* (Figs 4, 5).** Head capsule varies among individuals, ranging from yellow-brown with numerous small brown maculae (Fig. 4) to almost dark brown (Fig. 5); thoracic tergites and abdominal tergites I–VI with two dark-brown longitudinal stripes; membranous region of pro-, meso-, metanotum, and abdominal segments I–VI gray-brown (Fig. 4) to dark-gray-brown (Fig. 5), paler on both sides; abdominal tergite VII yellow-brown to gray-brown with two dark-brown stripes, and several small brown maculae; abdominal tergite VIII yellow-brown (Fig. 4) to brown (Fig. 5) with several small brown maculae.

***Body*.** Measurements and body shape ratios shown in Table 1.

***Head*.**HW/OCW = 1.80–1.90. A/HW = 0.91–0.98; A2/A1 = 0.78–0.88; A3/A1 = 0.50–0.54. MP3/MP2 = 1.24–1.29.

***Thorax*.** Spiracles present on mesosternum.

***Abdomen*.** Spiracles present on segments I–VII.

## ﻿Discussion

This study describes the larvae of Cybister (M.) brevis, and therefore larvae of two of the four subgenera of *Cybister* (i.e. *Melanectes* and *Cybister* s. str.) are now described in detail (Alarie et al. 2011; Watanabe and Hayashi 2024). Michat et al. (2015) reported 112 morphological characters and provided a data matrix that included the subgenera *Cybister* (*C. tripunctatus* (Olivier, 1795)) and *Melanectes* (*C. sugillatus* Erichson, 1834). Based on this information, as well as on the descriptions of C. (C.) lewisianus (Watanabe and Hayashi 2024) and C. (M.) brevis (this study), two morphological differences are worth noting between the subgenera *Cybister* and *Melanectes*. First, the notches between medial and lateral projections of frontoclypeus (character 6 in Michat et al. 2015) appear to be very narrow in *Cybister* (Alarie et al. 2011; Watanabe and Hayashi 2024), compared to wide to very wide in *Melanectes* (Michat et al. 2015; Fig. 6). Secondly, the egg bursters (character 7 in Michat et al. 2015) are found to be rounded in *Cybister* (Alarie et al. 2011; Watanabe and Hayashi 2024), compared to spiniform in *Melanectes* (Michat et al. 2015; Figs 6, 24). An interesting observation resulting from this study concerns the appearance of the lamellae clypeales, which appear to be drill-like in C. (C.) lewisianus (see Watanabe and Hayashi 2024: fig. 21), compared to having a weak longitudinal unevenness and split into several hair-like projections in C. (M.) brevis (Fig. 23). These morphological differences may help at distinguishing larvae of the subgenus Cybister from those of *Melanectes*. However, further research including additional species is needed to assert the diagnostic value of these characters.

## Supplementary Material

XML Treatment for Cybister (Melanectes) brevis

## ﻿Additional information

### Use of AI

No use of AI was reported.

### Author ORCIDs

Kohei Watanabe https://orcid.org/0000-0002-8761-232X

Masakazu Hayashi https://orcid.org/0000-0003-3708-9005

### Data availability

All of the data that support the findings of this study are available in the main text.
